# Global Sex Disparity of COVID-19: A Descriptive Review of Sex Hormones and Consideration for the Potential Therapeutic Use of Hormone Replacement Therapy in Older Adults

**DOI:** 10.14336/AD.2020.1211

**Published:** 2021-04-01

**Authors:** Samuel C Okpechi, Jordyn T Fong, Shawn S Gill, Jarrod C Harman, Tina H Nguyen, Queendaleen C Chukwurah, IfeanyiChukwu O Onor, Suresh K Alahari

**Affiliations:** ^1^Department of Biochemistry and Molecular Biology, Louisiana State University Health Sciences Center, New Orleans, LA 70112, USA.; ^2^Department of Biological Sciences, College of Science, Louisiana State University, Baton Rouge, LA 70803, USA.; ^3^Ochsner Clinical School, University of Queensland, Brisbane, Queensland, Australia.; ^4^Department of Ophthalmology, Louisiana State University Health Sciences Center, New Orleans, LA 70112, USA.; ^5^Neuroscience Center of Excellence, Louisiana State University Health Sciences Center, New Orleans, LA 70112, USA.; ^6^School of Medicine, Louisiana State University Health Sciences Center, New Orleans, LA 70112, USA.; ^7^Tulane Center for Aging, Tulane University School of Medicine, New Orleans, LA 70112, USA.; ^8^College of Pharmacy, Xavier University of Louisiana, New Orleans, LA 70125, USA.; ^9^Louisiana Cancer Research Center, Louisiana State University Health Sciences Center, New Orleans, LA 70112, USA.

**Keywords:** SARS-CoV-2, COVID-19, sexual disparity, sex hormones, older adults

## Abstract

The 2019-2020 SARS-related coronavirus-2 (SARS-CoV-2) pandemic has brought unprecedented challenges to healthcare sectors around the world. As of November 2020, there have been over 64 million confirmed cases and approaching 2 million deaths globally. Despite the large number of positive cases, there are very limited established standards of care and therapeutic options available. To date, there is still no Food and Drug Administration (FDA) approved vaccine for COVID-19, although there are several options in various clinical trial stages. Herein, we have performed a global review evaluating the roles of age and sex on COVID-19 hospitalizations, ICU admissions, deaths in hospitals, and deaths in nursing homes. We have identified a trend in which elderly and male patients are significantly affected by adverse outcomes. There is evidence suggesting that sex hormone levels can influence immune system function against SARS-CoV-2 infection, thus reducing the adverse effects of COVID-19. Since older adults have lower levels of these sex hormones, we therefore speculate, within rational scientific context, that sex steroids, such as estrogen and progesterone, needs further consideration for use as alternative therapeutic option for treating COVID-19 elderly patients. To our knowledge, this is the first comprehensive article evaluating the significance of sex hormones in COVID-19 outcomes in older adults.

## 1. Introduction

Amidst the SARS-related coronavirus-2 (SARS-CoV-2) pandemic, it is important to investigate vulnerable populations and possible treatment options. Despite public health campaigns to encourage social distancing, quarantining, hygiene practices, and wearing face masks, the number of cases of Coronavirus Disease 2019 (COVID-19) has yet to decline [1-3]. The virus was first identified in Wuhan, Hubei, People’s Republic of China in December 2019 when cases of unexplained pneumonia and acute respiratory distress syndrome (ARDS) were linked to an open-air seafood wholesale market. Scientists have proposed that the virus is transmitted to humans from the reservoir bats through different hosts [4]. By January 2020, the virus was only spreading within Wuhan; but since then, near global spread has occurred resulting in the World Health Organization (WHO) declaring a ‘pandemic’ on March 11th, 2020 [5].

The geriatric population is particularly vulnerable to suffering severe symptoms and mortality due to pre-existing comorbidities and diminished immunity [6, 7]. New data have revealed that men are at greater risk of suffering severe symptoms and mortality compared to women. According to information gathered from Global Health 50/50.org, the populations more likely to suffer severe symptoms exist at the intersection of old age and being male. There is speculation that the effect of sex steroid hormones may modestly contribute to this disparity of increased intensive care unit (ICU) admissions between men and women. Additionally, the natural waning of hormone production over the course of one’s lifetime can similarly account for the increased risk of death in the elderly population [8]. The correlation between sex hormones, disease severity, and death rates reveals the potential of utilizing hormone replacement therapy (HRT) to modulate the immune response to increase resilience to adverse disease outcomes [9, 10].

Nursing homes are well-documented settings for acquisition of nosocomial infection. Transmission of the SARS-CoV-2 virus through droplets makes it difficult to properly quarantine patients residing in these healthcare facilities [11]. Because COVID-19 has proven to be fatal particularly in the elderly population, it is necessary to consider possible agents that can minimize the bodily inflammatory responses that may lead to death. As previously stated, men are more likely than women to develop severe symptoms. One advantage that women have is higher levels of estrogen and progesterone, which has been shown to modulate a more robust immune response [12-15]. In order to decrease the incidence of infection and disease severity of COVID-19, HRT may be proposed as a method of boosting immunity of individuals who are most at risk. HRT has been used to alleviate symptoms of menopause and aging in both men and women, respectively. Currently there are two clinical studies investigating the effectiveness of estrogen and progesterone therapy to decrease disease severity of COVID-19 (Table 1). Overall, we propose that the clinical application of sex hormone therapy might be a viable alternative for the treatment of the adverse inflammatory effects of COVID-19 in older adults, especially males, as they are the most affected of both sexes.

**Table 1:** COVID-19 Clinical Trials Using Hormone Replacement Therapy (HRT).

Clinical Trial	Description	Intervention	Phase	Status	Eligibility	Participant #
Estrogen Patch for COVID-19 Symptoms Clinical Trial Identifier: NCT04359329	The use of an estrogen patch for 7 days could reduce the severity of COVID-19 symptoms. Determined by the rate of hospitalization, transfer to ICU, intubation, and death over 30 days.	Estradiol Patch. 100μg/day for 7 days	2	Recruiting	18+ males, 55+ female, COVID-19+, 1+ of the following symptoms; fever>100.5, SOB, cough, pneumonia+ x-ray	110
Progesterone for the Treatment of COVID-19 in Hospitalized Men Clinical Trial Identifier: NCT04365127	The administration of progesterone to determine the safety and efficacy in hospitalized COVID-19 positive males. Determined by the clinical status of subject at Day 15 and Day 29 and the duration of supplemental oxygen.	Progesterone 100MG. Injected twice daily for 5 days	1	Active, but not recruiting	18+ males, COVID-19+, Respiratory symptoms, Abnormal lung exam, Abnormal x-ray, ↓ O2saturation	40

## 2. SARS-CoV-2: Structure, mechanism of infection, and COVID-19 symptoms

SARS-CoV-2 is an enveloped, positive sense, single stranded RNA virus that is ~30 kb in length [4]. It is a member of the genus Betacoronavirus and the subgenus Sarbecovirus. Its genome codes for 4 structural proteins, one of which is the Spike (S) glycoprotein. S proteins are responsible for attracting and facilitating binding with human Angiotensin Converting Enzyme 2 (ACE-2) receptors, found in human respiratory epithelium and lung parenchyma, thus aiding viral entry [4]. ACE-2 is a key component to vasodilation via the Renin-Angiotensin-Aldosterone-System (RAAS) and is upregulated in commonly used pharmacotherapies for managing blood pressure. Because binding of the viral S protein to ACE-2 may increase the risk of infection, ACE inhibitors have been suggested as a possible therapy for COVID-19. Interestingly, it was recently reported that sex steroids can skew ACE-2 expression in human airway; therefore, it is plausible to believe that there is a relationship between the levels of endogenous sex hormones, ACE-2 expression, and the severity of SARS-CoV-2 infection. SARS-CoV-2 is the 7th virus of the family Coronaviridae (Coronaviruses) known to infect humans; four of them being typical causes of the common cold [16]. The SARS-CoV-2 virus is most similar to the Severe Acute Respiratory Syndrome Coronavirus (SARS-CoV), which caused the SARS pandemic from 2002-2003 [14].

In a study analyzing the viral genomic sequences of SARS-CoV and SARS-CoV-2, scientists noted a 79% similarity on a nucleotide level between them in the S protein region segment 601-640, including the D614 position [17]. Mutations of the viral genome have resulted in the emergence of distinct clades, whose variation may influence global disparities in infection rates. The initial outbreak in Wuhan was of Clade O, but the current dominant strain is thought to be the A2a variant. Unique mutations have been noted in studies conducted in several countries [18]. In terms of mutations in the A2a variant clade, a defining difference was observed in SARS-CoV-2 S protein aspartic acid D614 resulting in a shift to glycine, creating a D614G mutation [17]. Other mutations have also been noted to contribute to SARS-CoV-2 global diversity. For example, the P323L mutation of RNA-dependent RNA polymerase is hypothesized to be conserved during viral replication [18].

**Figure 1.**
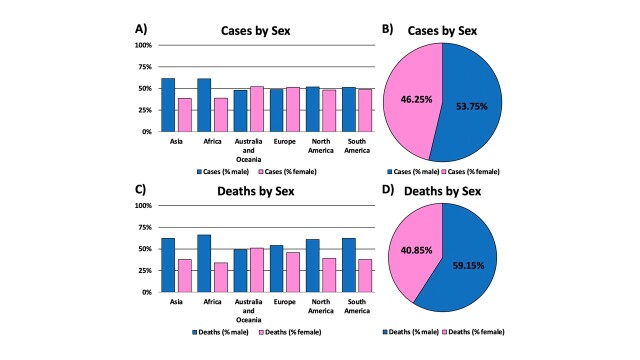
Global mortality and sexual disparity of COVID-19 by continents. (A) Global COVID-19 cases by sex and continent (B) Pie chart representation of the percentage of COVID-19 cases by sex. (C) Global mortality rate of COVID-19 deaths by sex and continent. (D) Pie chart representation of COVID-19 death percentages by sex. Male data is represented in blue, while female data is represented by pink. Overall, data suggests that males have an increased risk for COVID-19 infection and death, when compared to female counterparts. *Data was generated from Global Health 5050.org, on the 16th of November 2020.*

Human infection with SARS-CoV-2 is asymptomatic in many that are infected, aiding its ability to circulate undetected. In patients with severe disease acuity, the most common complications include pneumonia, respiratory failure, sepsis, cardiac injury, arrhythmia, kidney injury, metabolic acidosis, or hypoxic encephalopathy [19]. These complications can worsen the symptoms of COVID-19 and might also be the reason for high death rates in older adults.

## 3. COVID-19 global mortality rate and sex disparity

From the infancy of the COVID-19 pandemic, researchers worldwide observed a disparity of morbidity and mortality between men and women. Data suggest that men who contract the virus tend to have more severe symptoms compared to women, potentially inflating their death rate. This may be explained by differences in sex steroid levels which can alter the immune response, thus leading to a disparity in disease burden [13]. Although infection rates amongst men and women are marginal, men have a higher death rate than women by 18.3%, with men accounting for 59.15% of total deaths (Fig. 1). One proposed explanation is smoking, which is a habit more prevalent in men than in women [20]. SARS-CoV-2 is spread through and primarily affects the respiratory system, putting those who smoke at higher risk for severe complications. Smoking could exacerbate underlying conditions that would worsen symptoms and prognosis of COVID-19. This risk factor adversely affects outcomes in men.

Outbreaks of similar coronaviruses, SARS and MERS, provide a foundation from which researchers can reference. One SARS study that focused on the role of sex hormones on infection and mortality in mice demonstrated that male mice were highly susceptible to the infection despite being exposed to the same viral load. Male mice also experienced 90% mortality compared to 20% in female mice [12]. Researchers then compared gonadectomized mice to their counterparts after infection. The gonadectomized males did not show any difference in mortality or morbidity rate, but gonadectomized females had a significant increase in deaths [12]. These results suggest that testosterone may not play a role in infection or death rates; however, estrogen can influence both infection rates and disease severity.

The afore-mentioned researchers also studied the relationship of age on SARS mortality rates. They concluded that the young age group was most resistant to infection, and all mice in the oldest group died [12]. The CDC has published that the average age of patients who progressed to ARDS was 61 years old, and those who were spared were on average 49 years old [21].

**Figure 2.**
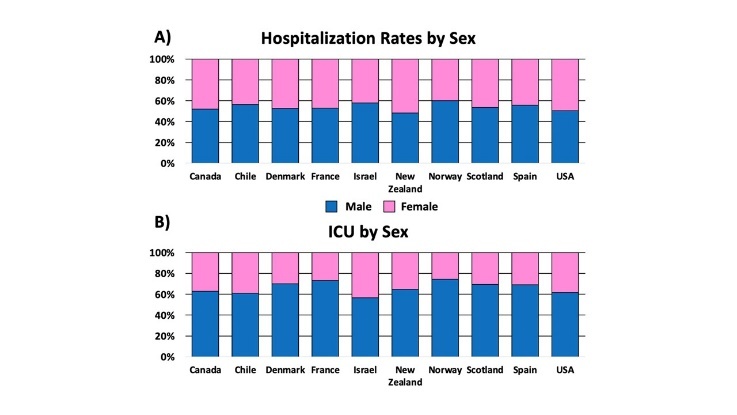
Sex-dependent effects on COVID-19 hospitalizations and ICU admission by select countries. (A) COVID-19 related hospitalizations; and (B) ICU admissions, by sex. Men and women are depicted by blue and pink datapoints, respectively. These charts reveal the overall male preponderance in COVID-19 hospitalization and ICU admission, with Norway exhibiting the largest sex-dependent predilection. The selected countries were chosen because they have publicly available data for hospitalizations and ICU admissions. *Data was generated from Global Health 5050.org, on the 16th of November 2020.*

## 4. COVID-19-related hospitalizations and ICU admissions by sex

Hospitalization and ICU admission rates by sex have recently become available for 10 countries: Canada, Chile, Denmark, France, Israel, New Zealand, Norway, Scotland, Spain, USA (Fig. 2). In these countries, males account for more than half of hospitalizations and ICU admissions. The largest sex difference observed occurred in Norway, with 60% of males being hospitalized and 74% of males in the ICU. This trend brings into perspective the sex disparity in how males and females respond to COVID-19. Further analysis of data set reveals that, though both sexes are susceptible to SARS-CoV-2 infection in the geriatric population, males are more likely to be admitted to the ICU (Fig. 2B).

## 5. Prevalence of COVID-19-related deaths in nursing homes

**Figure 3.**
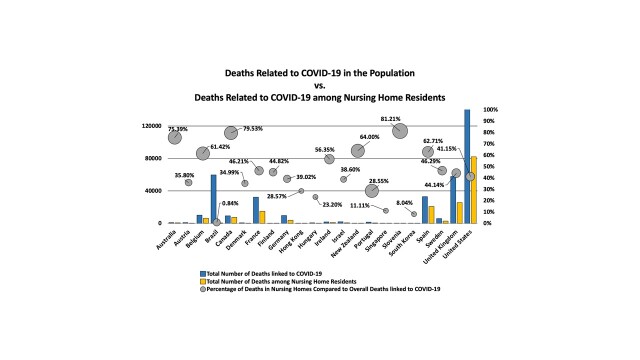
Total number of deaths in nursing homes linked to COVID-19. The United States has the highest rates of COVID-19 deaths in general. Overall, New Zealand saw a much lower total number of cases but observed the highest mortality rate within nursing homes when comparing deaths in nursing homes to total COVID-19 deaths. This figure highlights the relationship of COVID-19 related deaths (y-axis) from hospitals (blue bar graphs) to that of nursing homes (yellow bar graphs). The grey circles represent the percentage of nursing home deaths to the overall mortality rates linked to COVID-19. Countries are defined alphabetically along the x-axis.

Nursing homes are long-term care facilities that provide health care services to people who are not eligible for hospital admission but cannot be cared for at home. Between 2015 and 2016, American adults aged 65 years and older made up about 83.5% of the total nursing home patient population [22]. Infection in nursing home care facilities, especially among older adults, is a longstanding healthcare challenge. In the current COVID-19 infection pandemic, nursing homes have been severely hit with disproportionately higher rates of mortality than the general population (Fig. 3).

As of October 21st, 2020, international data from 22 countries reveal that 46-47% of the total mortality rate among nursing home residents is attributable to COVID-19 related deaths [23]. For this analysis, COVID-19 related death was defined as follows: i) death of a person who tests positive, before or after death, ii) death of a person who was clinically or epidemiologically suspected to have COVID-19, and iii) excess deaths when compared to the deaths that occurred in the same week for the previous year [23]. The countries with the highest number of home care residents’ COVID-19 deaths as a percentage of all COVID-19 deaths include Slovenia (81.2%), Canada (79.5%), and Australia (75.4%). Jordan and South Korea had less than 10% of COVID-related deaths in nursing homes [23].

It is important to consider that most of the nursing home residents affected by COVID-19 had common chronic underlying conditions including hypertension, cardiac disease, renal disease, diabetes, obesity, and pulmonary disease [24]. In terms of preventive measures against COVID-19 in nursing homes, the WHO published an interim COVID-19 prevention and control guidance for long-term care facilities on the 21st of March 2020 [25]. The main objective of the guideline is to prevent increases in new cases as well as horizontal spread of COVID-19 within and outside of nursing home facilities. Similarly, the CDC also developed a COVID-19 Infection Control Assessment and Response (ICAR) tool to help nursing homes and other long-term care facilities assess their infection prevention preparedness and quality improvement so that lapses identified can be specifically addressed [26].

## 6. Considerations for clinical and therapeutic use of sex steroid hormones

The three major sex steroid hormones are estrogen, progesterone, and testosterone. The hypothalamus-pituitary-gonadal (HPG) axis regulates the neuroendocrine feedback loop that facilitates steroidogenesis. These hormones are synthesized endogenously but their levels vary by sex and decrease with older age. Sex hormones play crucial regulatory roles in the process of human development and directly have an impact on human behavior, anatomy, and physiology. Testosterone is the dominant hormone in males and estrogen/progesterone are the dominant hormones in females. Both males and females go through a decrease in sex hormone production with age, but the rate at which the level of sex hormones decreases depends on the individual. As the female body undergoes menopause, the natural cessation of menses, the level of sex hormones in the body dramatically decreases compared to the male body, which tapers off slowly over time [27, 28]. Factors such as obesity and chronic liver disease can also affect the concentration of estrogen amongst individuals [28].

Exogenous HRT can replenish the low levels or loss of sex hormones in an individual. For example, HRT is common among post-menopausal women to mitigate symptoms of menopause, such as hot flashes, night sweats, mood swings, and vaginal atrophy [28]. Both males and females experience a weakening of bones, a condition known as osteoporosis, which can also be countered therapeutically using hormone replacement therapy [28, 29]. Testosterone replacement is often supplemented in men through a topical gel [27], but is also provided orally in pill form, or administered via intramuscular injection. Estrogen is administered to females as estradiol, a potent form of estrogen, which comes in different forms such as pills, patches, topical creams and suppositories [28]. Progesterone is usually taken as a pill alongside estrogen because it can reduce the risk of some cancers and is available in other formulations such as progestin IUD or progestin dermal implant [28]. Though sex steroids are useful for hormone-balance restoration in post-menopausal adult females and aged adult males, several groups of scientists have reported on additional interesting therapeutic benefits of sex steroid hormones including their roles in alleviating disease burden due to viral infections [10].

HRT has been associated with therapeutic benefits in improving patient outcomes in osteoporosis, cardiovascular disease, muscle mass, physical strength, and viral infections [8]. Potential risk factors of estrogen replacement therapy are breast and endometrial cancer [30]. However, there is no increased risk of developing cancer until after 15-20 years on estrogen replacement therapy (ERT) [30]. Trials were performed to further understand the risk and to evaluate ways to prevent malignancy; scientists concluded that using estrogen concurrently with progesterone can prevent osteoporosis without the increased risk of endometrial cancer but may increase risk for ischemic heart disease [30]. Although progesterone alone can have a preventative effect against endometrial cancer, its use should be carefully monitored because long term use of progesterone has been linked with the development of breast cancer [31].

In summary, HRT can be beneficial for both male and female geriatric patients who experience a natural decrease in sex hormone production. Not only does HRT mitigate undesirable side effects of aging such as decreased sex drive, hot flashes, and fatigue, but it can also reduce the risk of bone fractures and cardiac events. The focus of this review, however, is to assess the use of HRT to decrease disease burden in the geriatric population from SARS-CoV-2 virus infection. Such clinical trials investigating the efficacy of HRT for SARS-CoV-2 are short-term, thus cancer risk in women may be negligible if patients are being treated in the duration of a pandemic.

## 7. HRT to modulate immune response against SARS-CoV-2 infection

The two layering of the immunologic defense mechanisms of the body are the innate and adaptive subsystems [32]. The innate immunity is the first line of defense against SARS-CoV-2 infection. The main cellular components of the innate immune system include dendritic cells, monocytes, macrophages, granulocytes, and natural killer T cells [32]. However, when the acute response of the innate immunity is incapable of neutralizing and eliminating the invading virus, the adaptive immune system is mobilized to assist in combating the viral infection. The adaptive immune system is slow but specific, consisting of B and T cells [32, 33]. The adaptive immune system is known to respond to infections by using antigen receptor genes to recognize invading pathogenic antigens [32]. Researchers are investigating the crosstalk between sex hormones and the immune system regulation with the rationale that sex hormones can influence the immune system against SARS-CoV-2 infection. For example, elevated estrogen concentration and/or estrogen receptor alpha (ERα) activation have been shown to enhance type I and III interferon (IFN) synthesis, leading to a resultant decrease in virus titer [34].

After a viral infection, an increased number and activation of immune cells including lymphocytes, myeloid cells, neutrophil leukocytes, monocytes, alveolar macrophages, and dendritic cells are observed in circulation [34]. Clinical and biomedical studies have reported that sex hormones can influence the function of immune cells against viral infections through binding to specific receptors expressed in circulating macrophages, dendritic cells, lymphocytes, and in various lymphoid tissues [34-36]. Upon binding of hormones to the receptors on immune cells, a cascade of signaling pathways including c-Jun, NF-κB, and interferon regulatory factor (IRF) 1 are influenced, resulting in varying levels of cytokine and pro-inflammatory responses [34]. Activated ERα has been shown to inhibit NF-kB mediated inflammatory response and cytokine production through regulation of cells of the immune system including lymphocytes, macrophages, and neutrophils [37].

Cytokine storm and excessive inflammation are two major physiological events associated with SARS-CoV-2 viral infection [38-40]. The cytokine storm is usually associated with an inflammatory response to viral infections, triggering an increase in pro-inflammatory cytokine levels [41]. At normal physiological levels, sex steroid hormones, especially estrogen, have been reported to possess an anti-inflammatory effect, but this effect was seen only in pre-menopausal women [42-44]. However, it has been reported that though post-menopausal women have higher levels of pro-inflammatory cytokines, such as interleukin (IL)-6 and IL-1 (major cytokines involved in SARS-CoV-2 inflammatory process), the use of estrogen-associated hormone replacement therapy can decrease the levels of these cytokines to pre-menopausal levels [44]. Testosterone, the major male sex steroid hormone, have also been reported to efficiently downregulate inflammation through the increase of anti-inflammatory cytokines (IL-10) and the decrease of pro-inflammatory cytokines (IL-1β, IL-6, and TNF-α) [45, 46]. These studies suggest that sex hormones are viable candidates for consideration in the development of therapeutics against SARS-CoV-2 viral infection.

**Figure 4.**
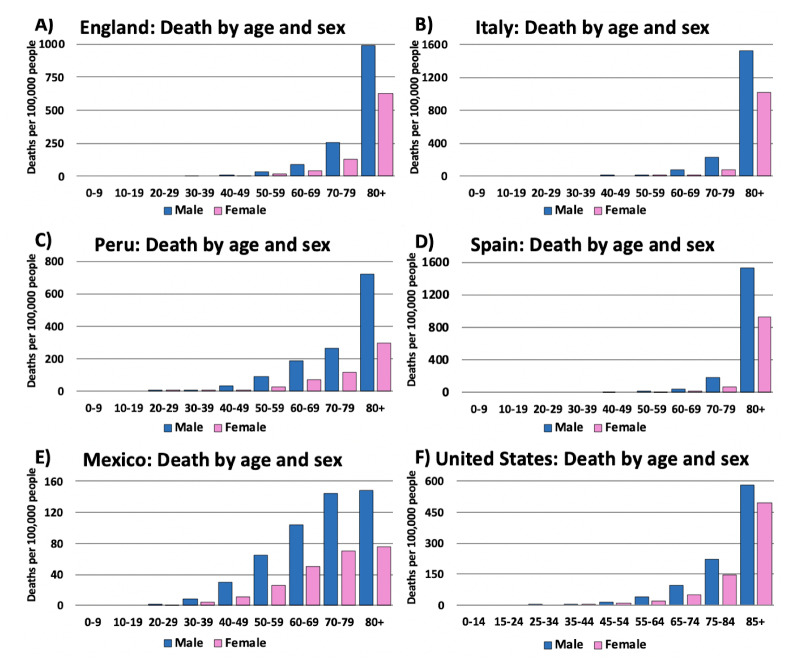

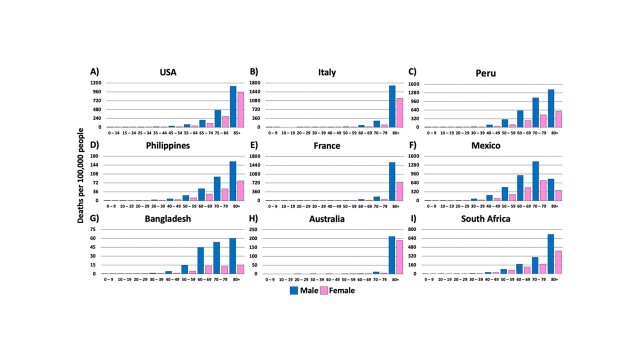
COVID-19 related deaths by age and sex in the top nine most affected countries: (A) USA (B) Italy (C) Peru (D) Philippines (E) France (F) Mexico (G) Bangladesh (H) Australia (I) South Africa. In all cases, there is a trend confirming that older adults experienced the highest death tolls from COVID-19. Additionally, in all cases herein, male patients exhibited higher mortality rates. In an effort to represent the global COVID-19 incidence and death rates, in each continent, we selected the highest-ranking country with publicly available data. It is worth noting that in Australia, France, and Italy, adults over the age of 80, especially males (B & E), were significantly more impacted when compared to additional age groups in the other countries. Male data is represented in blue, while female data is represented by pink. *Data was generated from Global Health 5050.org, on the 16th of November 2020.*

## 8. Effects of aging, sex, and associated changes in immune system components

The delicate balance between the innate and adaptive immune systems in response to pathogenic infections can be altered as age increases [47]. Not only does aging promote the chronic activation of the innate immune system and facilitates the decline of adaptive immune functions, it also causes a natural decline in sex hormone production [48-50]. Therefore, it is expected that females and males would differ in their susceptibility and response to SARS-CoV-2 infection, potentially explaining the obvious disparity in incidence, severity, and mortality rate. The activity of innate immune cells like macrophages and dendritic cells inducing inflammatory responses are generally higher post viral infection in females than in males, as demonstrated in several animal studies [51-53]. It is therefore plausible to conclude that the nature of immune response to SARS-CoV-2 infection can be altered by supplementing endogenous sex steroid hormones at various physiological stages, such as before and after menopause, during pregnancy, and during hormone replacement therapy [54]. The changes in immune components have been observed to show a bimodal sex hormone decline by age distribution. The first time point occurs in both men and women around 40 years of age, and the second time point occurs in men around the early/mid 60’s and women around the late 60’s [47].

In China, it was observed that there was a doubling in COVID-19 death rate in individuals around 40 years old from 0.2% to 0.4% (www.worldometers.info/coronavirus/coronavirus-age-sex-demographics/). A second wave of declining immunity occurs in individuals around 60 years which coincides with an increase in severity and lethality of COVID-19. This pattern is evident in the death rates reported in different countries of the world (Fig. 4). Interestingly, Italy, France, and Australia (Fig. 4B, 4E, and 4H, respectively) have responded to the pandemic effectively enough that the population ages 40-60 years are well protected. This is evident from the lower age-dependent incidence of COVID-19 cases. Worth noting, the mortality rate in males in Italy (Fig. 4B) and France (Fig. 4E) were significantly higher than males in other countries.

A recent study of 244 older COVID-19 patients reported that age and decreased lymphocyte count were independently associated with hospital mortality [55]. Another recent study has shown evidence that the timing of age-related changes in the immune system differ between men and women [56]. The authors reported that aging-related changes in both men and women included a decline in adaptive immunity, and an activation of innate immunity. These changes of increased inflammatory events were reported to be more devastating in men. According to their data, peripheral blood B lymphocytes were lower in older men aged 65+ years. This result corresponds with clinical study data generated from separate cohorts in France [56] and Japan [57]. Therefore, these lines of evidence suggest that sex-related changes in immunity can be altered by sex and with increase in age.

Presently, sex steroid hormones are among the list of potential therapies being considered for preventing and improving symptoms of COVID-19 disease. However, there is currently no published study evaluating the effect of HRT intervention for the different stages of COVID-19 in patients [58]. Hence, more biomedical and clinical translational studies are needed to better understand the pharmacological effects of exogenous hormone replacement dosing and duration that can alleviate COVID-19 incidence in patients.

## 9. Sex hormone therapy to prevent COVID-19 infection in the geriatric population

There is still a paucity of therapeutic options for treating COVID-19. Additionally, there are insufficient number of testing sites in many countries around the world, particularly in underdeveloped countries. Currently, physicians may only offer supportive care to patients who have severe symptoms and require hospitalization. In the most severe cases, remdesivir, a nucleoside analog, may be administered to patients [59]. Remdesivir is an antiviral drug that functions to inhibit the viral genome replication of SARS-CoV-2 [60]. While we are optimistic that remdesivir will have minimal long-term side effects, we also believe that exploring other potential alternatives for COVID-19 treatment would be in the best interest of the citizens of the world. We now know that sex hormones have other biological functions including antiviral effects. Therefore, it is rational to consider sex steroid hormones as potential treatment option in alleviating COVID-19 inflammatory and cytokine storm events, thereby improving quality of life for geriatric individuals, especially male counterparts.

### 9.1. Androgen (Testosterone)

It has been reported that androgen receptor activity is a requirement for the transcription of the transmembrane protease, serine 2 (TMPRSS2) gene [46, 61, 62], a cell surface protein that is expressed by epithelial cells of specific tissues including those in the aerodigestive tract [61]. Also, there is existing evidence that TMPRSS2 activity is necessary for the activation of the SARS-CoV-2 spike protein, viral entry, and spread in an infected host [61, 62]. Of note, SARS-CoV, the virus responsible for the 2003 SARS outbreak, H1N1 influenza, and the virus responsible for the 1918 and 2009 influenza pandemics, depend on TMPRSS2 for viral activation and cell entry [63-65]. These studies have a unifying conclusion that the upregulation of TMPRSS2 by androgen receptor activity could be the reason for the increased male predominance in deaths from COVID-19. Surprisingly, we have come to realize that this is not the universally accepted belief in the research field, many researchers maintain differing opinions.

A recent report has evidence that testosterone supplementation can reduce COVID-19 cytokine storm effect; thereby, suppressing SARS-CoV-2 induced inflammation [46]. In SARS-CoV-2 pneumonia patients, lower total testosterone (TT) was found in patients transferred to the ICU or deceased in the respiratory intensive care units (RICU) compared to patients transferred to internal medicine units or maintained in the RICU in stable condition [62]. Another group conducted a retrospective study where the investigators systematically analyzed sex hormones as well as cytokine and chemokine responses in male and female patients with laboratory-confirmed SARS-CoV-2 infections. They reported that a significant number of male COVID-19 patients had low testosterone (68.6%) and low dihydrotestosterone (48.6%) levels [66]. Though these studies were conducted in SARS-CoV-2 infected patients, we still believe that more studies are needed to validate the antiviral effect of testosterone. Presently, there are on-going COVID-19 clinical trials experimenting with progesterone and estrogen but there is yet to be a clinical trial investigating the use of testosterone therapy in COVID-19 patients.

### 9.2. Progesterone

Compared to estrogen and testosterone, there are fewer studies investigating the therapeutic potential of progesterone in reducing the disease burden of COVID-19 patients, particularly in older adults. Progesterone deserves more research for its possible antiviral effect against SARS-CoV-2 as there is existing evidence that progesterone has antiviral effects against HIV [67] and against influenza virus A [63]. Progesterone has also been reported to reduce inflammatory events at mucosal sites, like the respiratory tracts post-viral infection [68]. In 2010, a study reported that there was an observable decrease in progesterone levels in SARS patients compared to healthy controls. This implies that progesterone signaling pathway might be involved in antiviral immune response.

More recently, a study published in *Nature* reports that progesterone has an anti-SARS-CoV-2 effect [69]. Another group reported that, in their study, four out of eight pregnant women infected with SARS-CoV-2 were asymptomatic before delivery but became symptomatic post-partum [70] further suggesting that progesterone could be protective against SARS-CoV-2 infection. This finding further strengthens the case that progesterone, which is elevated during pregnancy and abruptly drops upon delivery, might have potent anti-viral properties. Furthermore, another recent study has also reported that progesterone, in addition to estrogen and testosterone, has anti-SARS-CoV-2 effect notably in older adults [71]. Presently, there is an ongoing clinical trial [NCT04365127] that seeks to investigate the safety and efficacy of progesterone for treatment of COVID-19 in hospitalized men (Table 1). The findings from this clinical trial will strengthen or weaken the existing evidence that progesterone has antiviral activity against SARS-CoV-2.

### 9.3. Estrogen

Estrogen is the most studied sex steroid hormone because of its many therapeutic uses, especially in combating viral infections. Estrogen (17β-estradiol) has been reported to have antiviral effects against viruses including influenza virus [72], hepatitis C virus [73], SARS [74], and SARS-CoV-2 [13, 71]. Estrogen and its immunomodulating effects against SARS-CoV-2 infection is presently under active investigation in different laboratories around the world. Very recently, estrogen was shown to possess anti-SARS-CoV-2 effect which occurs through the suppression of TMPRSS2 expression in different cell lines [13]. The antiviral effect of estrogen is due, in part, to the many antiviral genes or inflammatory genes that have estrogen response elements (EREs) in their promoter regions which enhances estrogen-mediated cellular signaling [13].

In 2013, Hilliard *et al*. reported that estrogen-induced expression of ACE-2 is reduced in postmenopausal women [75]. This study, again, brings to the forefront a relevant clue why elderly women are likely less susceptible to SARS-CoV-2 infection than elderly men. According to Suba *et al,* Premarin, can have gene repairing power like endogenous estrogen without risk of adverse reactions [14]. Premarin is a conjugated equine estrogen derived from natural sources and has been reported to have proven antiviral efficacy [14]. Presently, there is an ongoing clinical trial [NCT04359329] that seeks to investigate if estrogen, given as a patch placed on the skin of COVID-19 patients including older adults can reduce the severity of COVID-19 symptoms compared to regular care (Table 1). Again, the findings from this clinical trial will strengthen or weaken the existing evidence that estrogen has antiviral activity against SARS-CoV-2.

## 10. Conclusion

The global burden of COVID-19 is taking a toll on financial, economic, and healthcare sectors of many countries around the world. This comprehensive review seeks to report, using a biomedical and clinical knowledge base, on one of the controversial topics that is worth further investigation at this critical time. Sex hormones are produced endogenously; therefore, they are not harmful to the body or possess adverse effects if their normal levels are maintained. However, the signaling pathways or events related to gene transcription might be altered leading to modified levels of these hormones which might then pose some physiological consequences. Here, we have summarized the perspectives of several endocrine, aging, and immunology scientists on the subject matter of sex hormones and how they regulate immune function against SARS-CoV-2 infection.

Here we have gathered and analyzed data sets that are available to the public to investigate the prevalence of COVID-19 related cases and deaths by continents. We then repeated the same process for COVID-19 related hospitalizations and ICU admissions in top countries with high COVID-19 prevalence. Finally, we analyzed death rates by sex and age to further strengthen our hypothesis that men are globally more affected by COVID-19 infection than women. Herein, we have summarized the main highlights and conclusions of relevant scientific and clinical studies published on sex hormone functions in immunity, aging, and against SARS-CoV-2 infection. There are now multiple sources of evidence that show how sex hormones can be used as viable candidates for consideration as potential antiviral prophylaxis. However, it should be noted that HRT could have some side effects such as breast and endometrial cancer in women.

In conclusion, compared to women, men are at higher risk of SARS-CoV-2 infection, ICU admission, hospitalization, and death rate in countries around the world. This disparity in COVID-19 pandemic is most likely multifaceted (biological, lifestyle, and comorbidities, etc.) but sex hormone levels are arguably one of the primary reasons why men and women respond differently to SARS-CoV-2 infection. As the virus continues to evolve and spread, there is an urgent need for more treatment options and therapeutic modalities that will be aimed at neutralizing SARS-CoV-2 infection and improving symptoms of the COVID-19 disease. Therefore, we recommend that the use of sex hormone as possible therapy for COVID-19 older adults be given maximum consideration as this clinical intervention might help reduce the global disease burden of COVID-19.

## References

[1] Gandhi M, Havlir D 2020. The Time for Universal Masking of the Public for Coronavirus Disease 2019 Is Now. In Open Forum Infectious Diseases: Oxford University Press US. ofaa131. 10.1093/ofid/ofaa131 PMC7179801 32346544

[2] GÜNER HR, HASANOĞLU İ, AKTAŞ F (2020). COVID-19: Prevention and control measures in community. Turkish Journal of medical sciences, 50:571-577. 32293835 10.3906/sag-2004-146 PMC7195988

[3] MacIntyre CR, Wang Q (2020). Physical distancing, face masks, and eye protection for prevention of COVID-19. The Lancet, 395(10242):1950-1951 10.1016/S0140-6736(20)31183-1 PMC7263820 32497511

[4] Jia HP, Look DC, Shi L, Hickey M, Pewe L, Netland J, et al. (2005). ACE2 receptor expression and severe acute respiratory syndrome coronavirus infection depend on differentiation of human airway epithelia. Journal of virology, 79:14614-14621. 16282461 10.1128/JVI.79.23.14614-14621.2005 PMC1287568

[5] South AM, Diz DI, Chappell MC (2020). COVID-19, ACE2, and the cardiovascular consequences. Am J Physiol Heart Circ Physiol, 1;318(5):H1084-H1090. 32228252 10.1152/ajpheart.00217.2020 PMC7191628

[6] Montecino-Rodriguez E, Berent-Maoz B, Dorshkind K (2013). Causes, consequences, and reversal of immune system aging. The Journal of clinical investigation, 123:958-965. 23454758 10.1172/JCI64096 PMC3582124

[7] Richardson S, Hirsch JS, Narasimhan M, Crawford JM, McGinn T, Davidson KW, et al. (2020). Presenting characteristics, comorbidities, and outcomes among 5700 patients hospitalized with COVID-19 in the New York City area. JAMA, 323(20):2098. 32320003 10.1001/jama.2020.6775 PMC7177629

[8] Horstman AM, Dillon EL, Urban RJ, Sheffield-Moore M (2012). The role of androgens and estrogens on healthy aging and longevity. Journals of Gerontology Series A: Biomedical Sciences and Medical Sciences, 67:1140-1152. 10.1093/gerona/gls068 PMC3636678 22451474

[9] Porter V, Greendale G, Schocken M, Zhu X, Effros R (2001). Immune effects of hormone replacement therapy in post-menopausal women. Experimental gerontology, 36:311-326. 11226745 10.1016/s0531-5565(00)00195-9

[10] Taneja V (2018). Sex hormones determine immune response. Frontiers in immunology, 9:1931. 30210492 10.3389/fimmu.2018.01931 PMC6119719

[11] Jayaweera M, Perera H, Gunawardana B, Manatunge J (2020). Transmission of COVID-19 virus by droplets and aerosols: A critical review on the unresolved dichotomy. Environmental Research:109819. 32569870 10.1016/j.envres.2020.109819 PMC7293495

[12] Channappanavar R, Fett C, Mack M, Ten Eyck PP, Meyerholz DK, Perlman S (2017). Sex-based differences in susceptibility to severe acute respiratory syndrome coronavirus infection. The Journal of Immunology, 198:4046-4053. 28373583 10.4049/jimmunol.1601896 PMC5450662

[13] Breithaupt-Faloppa AC, Correia CdJ, Prado CM, Stilhano RS, Ureshino RP, Moreira LFP (2020). 17β-Estradiol, a potential ally to alleviate SARS-CoV-2 infection. Clinics, 75:e1980. 32490931 10.6061/clinics/2020/e1980 PMC7233687

[14] Suba Z (2020). Prevention and therapy of COVID-19 via exogenous estrogen treatment for both male and female patients. Journal of Pharmacy & Pharmaceutical Sciences, 23:75-85. 32324533 10.18433/jpps31069

[15] Mauvais-Jarvis F, Klein SL, Levin ER (2020). Estradiol, progesterone, immunomodulation, and COVID-19 outcomes. Endocrinology, 161:bqaa127. 32730568 10.1210/endocr/bqaa127 PMC7438701

[16] Zhu N, Zhang D, Wang W, Li X, Yang B, Song J, et al. (2019). A novel coronavirus from patients with pneumonia in China, 2019. New England Journal of Medicine, 382 (8) (2020) 727-733. 10.1056/NEJMoa2001017 PMC7092803 31978945

[17] Koyama T, Weeraratne D, Snowdon JL, Parida L (2020). Emergence of drift variants that may affect COVID-19 vaccine development and antibody treatment. Pathogens, 9:324. 10.3390/pathogens9050324 PMC7281497 32357545

[18] Maitra A, Sarkar MC, Raheja H, Biswas NK, Chakraborti S, Singh AK, et al. (2020). Mutations in SARS-CoV-2 viral RNA identified in Eastern India: Possible implications for the ongoing outbreak in India and impact on viral structure and host susceptibility. J Biosci. 2020;45(1):76. 10.1007/s12038-020-00046-1 PMC7269891 32515358

[19] Baj J, Karakuła-Juchnowicz H, Teresiński G, Buszewicz G, Ciesielka M, Sitarz E, et al. (2020). COVID-19: Specific and Non-Specific Clinical Manifestations and Symptoms: The Current State of Knowledge. Journal of Clinical Medicine, 9:1753. 10.3390/jcm9061753 PMC7356953 32516940

[20] Walter LA, McGregor AJ (2020). Sex-and Gender-specific Observations and Implications for COVID-19. Western Journal of Emergency Medicine, 21:507. 10.5811/westjem.2020.4.47536 PMC7234726 32302282

[21] Surveillances V (2020). The epidemiological characteristics of an outbreak of 2019 novel coronavirus diseases (COVID-19)—China, 2020. China CDC Weekly, 2:113-122. PMC8392929 34594836

[22] Harris-Kojetin L, Sengupta M, Park-Lee E, Valverde R, Caffrey C, Rome V, et al. (2016). Long-term care providers and services users in the United States: data from the National Study of Long-Term Care Providers, 2013-2014. Vital & health statistics. Series 3, Analytical and epidemiological studies:x-xii; 1. 27023287

[23] Comas-Herrera A, Zalakaín J, Litwin C, Hsu AT, Lane N, Fernández J-L (2020). Mortality associated with COVID-19 outbreaks in care homes: early international evidence. rticle in LTCcovid.org, International Long-Term Care Policy Network, CPEC-LSE, 14 10.

[24] McMichael T, Clark S, Pogosjans S, Kay M, Lewis J, Baer A, et al. (2020). Public Health-Seattle & King County; EvergreenHealth; CDC COVID-19 Investigation Team. COVID-19 in a long-term care facility—King County, Washington, February 27-March 9, 2020. MMWR Morb Mortal Wkly Rep, 69:339-342. 32214083 10.15585/mmwr.mm6912e1 PMC7725515

[25] Organization WH. 2020. Infection prevention and control guidance for long-term care facilities in the context of COVID-19: interim guidance, 21 March 2020. World Health Organization.

[26] Telford CT, Onwubiko U, Holland DP, Turner K, Prieto J, Smith S, et al. (2020). Preventing COVID-19 Outbreaks in Long-Term Care Facilities Through Preemptive Testing of Residents and Staff Members—Fulton County, Georgia, March-May 2020. Morbidity and Mortality Weekly Report, 69:1296. 32941413 10.15585/mmwr.mm6937a4 PMC7498169

[27] Snyder PJ, Bhasin S, Cunningham GR, Matsumoto AM, Stephens-Shields AJ, Cauley JA, et al. (2016). Effects of testosterone treatment in older men. New England Journal of Medicine, 374:611-624. 10.1056/NEJMoa1506119 PMC5209754 26886521

[28] Fait T (2019). Menopause hormone therapy: latest developments and clinical practice. Drugs Context. 2019;8:212551. 10.7573/dic.212551 PMC6317580 30636965

[29] Stanworth RD, Jones TH (2008). Testosterone for the aging male; current evidence and recommended practice. Clinical Interventions in Aging, 3:25. 18488876 10.2147/cia.s190 PMC2544367

[30] Biscup P (2003). Risks and benefits of long-term hormone replacement therapy. American journal of health-system pharmacy, 60:1419-1425. 12892026 10.1093/ajhp/60.14.1419

[31] Regidor P-A (2014). Progesterone in peri-and postmenopause: a review. Geburtshilfe und Frauenheilkunde, 74:995. 25484373 10.1055/s-0034-1383297 PMC4245250

[32] Clark R, Kupper T (2005). Old meets new: the interaction between innate and adaptive immunity. Journal of Investigative Dermatology, 125:629-637. 10.1111/j.0022-202X.2005.23856.x 16185260

[33] Medzhitov R, Janeway CA Jr (2002). Innate immune recognition. Annu Rev Immunol, 20:197-216. 11861602 10.1146/annurev.immunol.20.083001.084359

[34] Kadel S, Kovats S (2018). Sex hormones regulate innate immune cells and promote sex differences in respiratory virus infection. Frontiers in Immunology, 9:1653. 30079065 10.3389/fimmu.2018.01653 PMC6062604

[35] Klein SL, Marriott I, Fish EN (2015). Sex-based differences in immune function and responses to vaccination. Transactions of the Royal Society of Tropical Medicine and Hygiene, 109:9-15. 25573105 10.1093/trstmh/tru167 PMC4447843

[36] Gebhard C, Regitz-Zagrosek V, Neuhauser HK, Morgan R, Klein SL (2020). Impact of sex and gender on COVID-19 outcomes in Europe. Biology of Sex Differences, 11:1-13. 32450906 10.1186/s13293-020-00304-9 PMC7247289

[37] Biswas DK, Singh S, Shi Q, Pardee AB, Iglehart JD (2005). Crossroads of estrogen receptor and NF-κB signaling. Science's STKE, 2005:pe27-pe27. 10.1126/stke.2882005pe27 15956359

[38] Al-Lami RA, Urban RJ, Volpi E, Algburi AM, Baillargeon J 2020. Sex Hormones and Novel Corona Virus Infectious Disease (COVID-19). In Mayo Clinic Proceedings: Elsevier. 10.1016/j.mayocp.2020.05.013 PMC7256539 32753145

[39] Conti P, Ronconi G, Caraffa A, Gallenga C, Ross R, Frydas I, et al. (2020). Induction of pro-inflammatory cytokines (IL-1 and IL-6) and lung inflammation by Coronavirus-19 (COVI-19 or SARS-CoV-2): anti-inflammatory strategies. J Biol Regul Homeost Agents, 34:1. 10.23812/CONTI-E 32171193

[40] Wadman M, Couzin-Frankel J, Kaiser J, Matacic C 2020. A rampage through the body. American Association for the Advancement of Science. 10.1126/science.368.6489.356 32327580

[41] Guo G, Ye L, Pan K, Chen Y, Xing D, Yan K, et al. (2020). New Insights of Emerging SARS-CoV-2: Epidemiology, Etiology, Clinical Features, Clinical Treatment, and Prevention. Frontiers in Cell and Developmental Biology, 8:410. 32574318 10.3389/fcell.2020.00410 PMC7256189

[42] Straub RH (2007). The complex role of estrogens in inflammation. Endocrine reviews, 28:521-574. 17640948 10.1210/er.2007-0001

[43] Gaskins AJ, Wilchesky M, Mumford SL, Whitcomb BW, Browne RW, Wactawski-Wende J, et al. (2012). Endogenous reproductive hormones and C-reactive protein across the menstrual cycle: the BioCycle Study. American journal of epidemiology, 175:423-431. 22306563 10.1093/aje/kwr343 PMC3282877

[44] Giefing-Kröll C, Berger P, Lepperdinger G, Grubeck-Loebenstein B (2015). How sex and age affect immune responses, susceptibility to infections, and response to vaccination. Aging cell, 14:309-321. 25720438 10.1111/acel.12326 PMC4406660

[45] Mohamad N-V, Wong SK, Hasan WNW, Jolly JJ, Nur-Farhana MF, Ima-Nirwana S, et al. (2018). The relationship between circulating testosterone and inflammatory cytokines in men. Aging Male, 22(2): 129-140. 29925283 10.1080/13685538.2018.1482487

[46] Pozzilli P, Lenzi A (2020). Commentary: Testosterone, a key hormone in the context of COVID-19 pandemic. Metabolism, 108:154252. 32353355 10.1016/j.metabol.2020.154252 PMC7185012

[47] Márquez EJ, Trowbridge J, Kuchel GA, Banchereau J, Ucar D (2020). The lethal sex gap: COVID-19. Immunity & Ageing, 17:1-8. 32457811 10.1186/s12979-020-00183-z PMC7240166

[48] Nikolich-Ž;ugich J (2005). T cell aging: naive but not young. The Journal of experimental medicine, 201:837-840. 15781575 10.1084/jem.20050341 PMC2213096

[49] Goronzy JJ, Weyand CM (2017). Successful and maladaptive T cell aging. Immunity, 46:364-378. 28329703 10.1016/j.immuni.2017.03.010 PMC5433436

[50] Fulop T, Larbi A, Dupuis G, Le Page A, Frost EH, Cohen AA, et al. (2018). Immunosenescence and inflamm-aging as two sides of the same coin: friends or foes? Frontiers in immunology, 8:1960. 29375577 10.3389/fimmu.2017.01960 PMC5767595

[51] Boissier J, Chlichlia K, Digon Y, Ruppel A, Mone H (2003). Preliminary study on sex-related inflammatory reactions in mice infected with Schistosoma mansoni. Parasitology research, 91:144-150. 12910415 10.1007/s00436-003-0943-1

[52] Xia H-J, Zhang G-H, Wang R-R, Zheng Y-T (2009). The influence of age and sex on the cell counts of peripheral blood leukocyte subpopulations in Chinese rhesus macaques. Cellular & molecular immunology, 6:433-440. 20003819 10.1038/cmi.2009.55 PMC4003037

[53] Melgert BN, Oriss TB, Qi Z, Dixon-McCarthy B, Geerlings M, Hylkema MN, et al. (2010). Macrophages: regulators of sex differences in asthma? American journal of respiratory cell and molecular biology, 42:595-603. 19574533 10.1165/rcmb.2009-0016OC PMC2874445

[54] Klein SL 2013. Sex differences in prophylaxis and therapeutic treatments for viral diseases. In Sex and Gender Differences in Pharmacology: Springer. 499-522. 10.1007/978-3-642-30726-3_22 23027464

[55] Sun H, Ning R, Tao Y, Yu C, Deng X, Zhao C, et al. (2020). Risk factors for mortality in 244 older adults with COVID-19 in Wuhan, China: a retrospective study. J Am Geriatr Soc, 68(6): E19-E23. 32383809 10.1111/jgs.16533 PMC7267277

[56] Piasecka B, Duffy D, Urrutia A, Quach H, Patin E, Posseme C, et al. (2018). Distinctive roles of age, sex, and genetics in shaping transcriptional variation of human immune responses to microbial challenges. Proceedings of the National Academy of Sciences, 115:E488-E497. 10.1073/pnas.1714765115 PMC5776984 29282317

[57] Hirokawa K, Utsuyama M, Hayashi Y, Kitagawa M, Makinodan T, Fulop T (2013). Slower immune system aging in women versus men in the Japanese population. Immunity & Ageing, 10:1-9. 23675689 10.1186/1742-4933-10-19 PMC3663722

[58] Cattrini C, Bersanelli M, Latocca MM, Conte B, Vallome G, Boccardo F (2020). Sex hormones and hormone therapy during covid-19 pandemic: Implications for patients with cancer. Cancers, 12:2325. 10.3390/cancers12082325 PMC7464909 32824674

[59] Control CfD, Prevention. 2020. Interim Clinical Guidance for Management of Patients with Confirmed 2019 Novel Coronavirus (2019-nCoV) Infection, Updated February 12, 2020.

[60] Eastman RT, Roth JS, Brimacombe KR, Simeonov A, Shen M, Patnaik S, et al. (2020). Remdesivir: A Review of Its Discovery and Development Leading to Emergency Use Authorization for Treatment of COVID-19. ACS Cent Sci, 6(6):1009 32483554 10.1021/acscentsci.0c00489 PMC7202249

[61] Stopsack KH, Mucci LA, Antonarakis ES, Nelson PS, Kantoff PW (2020). TMPRSS2 and COVID-19: Serendipity or Opportunity for Intervention? Cancer discovery, 10:779-782. 32276929 10.1158/2159-8290.CD-20-0451 PMC7437472

[62] Wambier CG, Goren A, Vaño-Galván S, Ramos PM, Ossimetha A, Nau G, et al. (2020). Androgen sensitivity gateway to COVID-19 disease severity. Drug Dev Res, 81(7):771-776. 32412125 10.1002/ddr.21688 PMC7273095

[63] Chaipan C, Kobasa D, Bertram S, Glowacka I, Steffen I, Tsegaye TS, et al. (2009). Proteolytic activation of the 1918 influenza virus hemagglutinin. Journal of virology, 83:3200-3211. 19158246 10.1128/JVI.02205-08 PMC2655587

[64] Matsuyama S, Nagata N, Shirato K, Kawase M, Takeda M, Taguchi F (2010). Efficient activation of the severe acute respiratory syndrome coronavirus spike protein by the transmembrane protease TMPRSS2. Journal of virology, 84:12658-12664. 20926566 10.1128/JVI.01542-10 PMC3004351

[65] Hoffmann M, Kleine-Weber H, Schroeder S, Krüger N, Herrler T, Erichsen S, et al. (2020). SARS-CoV-2 cell entry depends on ACE2 and TMPRSS2 and is blocked by a clinically proven protease inhibitor. Cell, 181(2):271-280.e8. 32142651 10.1016/j.cell.2020.02.052 PMC7102627

[66] Rastrelli G, Di Stasi V, Inglese F, Beccaria M, Garuti M, Di Costanzo D, et al. (2020). Low testosterone levels predict clinical adverse outcomes in SARS-CoV-2 pneumonia patients. Andrology, in press. 10.1111/andr.12821 PMC7280645 32436355

[67] Chapuy-Regaud S, Subra C, Requena M, de Medina P, Amara S, Delton-Vandenbroucke I, et al. (2013). Progesterone and a phospholipase inhibitor increase the endosomal bis (monoacylglycero) phosphate content and block HIV viral particle intercellular transmission. Biochimie, 95:1677-1688. 23774297 10.1016/j.biochi.2013.05.019

[68] Hall OJ, Klein SL (2017). Progesterone-based compounds affect immune responses and susceptibility to infections at diverse mucosal sites. Mucosal Immunology, 10:1097-1107. 28401937 10.1038/mi.2017.35

[69] Gordon D, Jang M 2020. G.; Bouhaddou, M.; Krogan, NJ A SARS-CoV-2-Human Protein-Protein Interaction Map Reveals Drug Targets and Potential Potential Drug-Repurposing. BioXriv.

[70] Wu C, Yang W, Wu X, Zhang T, Zhao Y, Ren W, et al. (2020). Clinical Manifestation and Laboratory Characteristics of SARS-CoV-2 Infection in Pregnant Women. Virologica Sinica:1-6. 10.1007/s12250-020-00227-0 PMC7167538 32314274

[71] Elfiky AA (2020). Natural products may interfere with SARS-CoV-2 attachment to the host cell. Journal of Biomolecular Structure and Dynamics:1-10. 10.1080/07391102.2020.1761881 PMC7212544 32340551

[72] Robinson DP, Lorenzo ME, Jian W, Klein SL (2011). Elevated 17β-estradiol protects females from influenza A virus pathogenesis by suppressing inflammatory responses. PLoS Pathog, 7:e1002149. 21829352 10.1371/journal.ppat.1002149 PMC3145801

[73] Magri A, Barbaglia MN, Foglia CZ, Boccato E, Burlone ME, Cole S, et al. (2017). 17, β-estradiol inhibits hepatitis C virus mainly by interference with the release phase of its life cycle. Liver International, 37:669-677. 27885811 10.1111/liv.13303 PMC5448036

[74] Wei L, Sun S, Zhang J, Zhu H, Xu Y, Ma Q, et al. (2010). Endocrine cells of the adenohypophysis in severe acute respiratory syndrome (SARS). Biochemistry and Cell Biology, 88:723-730. 20651845 10.1139/O10-022

[75] Hilliard LM, Sampson AK, Brown RD, Denton KM (2013). The “his and hers” of the renin-angiotensin system. Current hypertension reports, 15:71-79. 23180053 10.1007/s11906-012-0319-y

